# Improving the Use of Treatment Escalation Plans in Geriatric Medicine: A Quality Improvement Project

**DOI:** 10.7759/cureus.95283

**Published:** 2025-10-24

**Authors:** Jien Hwa Choo, Rachel Dee, Morven McElroy

**Affiliations:** 1 Department of Medicine for the Elderly, Glasgow Royal Infirmary, Glasgow, GBR

**Keywords:** dnacpr, geriatric medicine, palliative care, quality improvement, treatment escalation plan

## Abstract

Background

Treatment escalation plans (TEPs) support patient-centered decision-making by documenting individualized goals of care during clinical deterioration. Despite national guidance, TEP completion remains inconsistent, particularly in geriatric medicine, where frailty and multimorbidity complicate escalation decisions. Our audit aims to improve the uptake and timeliness of TEP completion in geriatric medicine wards of a tertiary hospital in Glasgow through targeted quality improvement interventions.

Methodology

A two-cycle quality improvement project was conducted between April and August 2025. Only patients admitted during the study periods and meeting predefined TEP criteria were included. Interventions included a paper-based admission checklist (Cycle 1) and a multidisciplinary teaching program (Cycle 2). Outcome measures included TEP completion rates, time to documentation, decisions regarding Do Not Attempt Cardiopulmonary Resuscitation (DNACPR), and consultant involvement.

Results

A total of 275 patients were included (baseline: 75; Cycle 1: 75; Cycle 2: 125). TEP completion rates improved from 21 of 75 patients (28%) at baseline to 32 of 75 (43%) in Cycle 1 and 72 of 125 (58%) in Cycle 2. Median time to TEP documentation decreased from 10.5 days (interquartile range (IQR) 7-14) to 5 days (IQR 3-8). DNACPR documentation increased from 55% to 73%, with a significant association between DNACPR completion and TEP completion (*P* = 0.01). Consultant involvement increased from 12% at baseline to 27% and was associated with earlier TEP completion (median 4 vs. 7 days, *P* = 0.03).

Conclusions

This audit demonstrated an initial approach to improving TEP uptake and timeliness in geriatric medicine. While targeted interventions showed measurable benefits, formal TEP education and ongoing strategies are required to support sustainable use and strengthen patient-centered decision-making.

## Introduction

A treatment escalation plan (TEP) is a clinical framework used to document and communicate individualized goals of care in the event of patient deterioration, ensuring that interventions align with the patient’s values, preferences, and clinical status. Beyond guiding real-time clinical decisions, TEP serves as a legal and ethical instrument that upholds patient autonomy [1]. Despite national guidelines advocating their use, significant variability persists in TEP completion rates, timing, and quality as demonstrated by audits both locally and nationally [2,3]. Studies have highlighted that this inconsistency is not confined to one setting; implementation of TEPs and similar constructs across acute hospitals also shows wide variation, with many plans incomplete or lacking senior input [4,5].

Evidence suggests that quality improvement (QI) interventions, such as education and workflow prompts, can significantly enhance the completion rates and timeliness of TEP documentation [6]. For example, Chua et al. demonstrated that structured initiatives in old age psychiatry inpatient services improved TEP use [7], while similar efforts in acute hospitals have reported measurable increases in compliance. However, it is increasingly recognized that quantity alone is insufficient; the quality and depth of TEP conversations and documentation are equally critical. Evaluations using frameworks of accountability have shown variable adherence to standards of reasonableness and patient-centeredness [8], while recent studies stress that well-completed TEPs can facilitate person-centered care and reduce decisional conflict [9].

This quality improvement project was designed to evaluate the baseline use of TEP in geriatric medicine wards within a tertiary hospital in Glasgow and to implement structured interventions aimed at improving their uptake and quality. Using two Plan-Do-Study-Act (PDSA) cycles, we aimed to improve documentation rates, decision-making quality, and multidisciplinary communication around treatment escalation in frail older inpatients.

## Materials and methods

Study design and setting

This quality improvement project was conducted within the geriatric medicine wards of a large tertiary teaching hospital in Glasgow, Scotland. The project employed a two-cycle PDSA methodology to evaluate and improve the utilization of TEPs. The TEP form is a standardized document already embedded in routine practice within the health board, designed to guide and record clinical decision-making about the appropriate level of medical intervention in the event of patient deterioration. Patients considered for a TEP typically meet one or more of the following clinical criteria:

● Any patient who has/will have a Do Not Attempt Cardiopulmonary Resuscitation (DNACPR) form

● Risk of deterioration or instability

● Frailty, completely dependent for Activities of Daily Living (ADLs)

● Progressive organ failure with or without multiple comorbidities

● Advanced cancer

● At the request of the patient/welfare attorney or guardian/nearest relative, or carers

Inclusion criteria

Only patients aged 65 and above admitted during the respective study periods to non-acute geriatric wards were included in the dataset. Patients not meeting the above criteria or whose notes were incomplete were excluded, e.g., incomplete DNACPR or TEP form, or missing notes. 

Data collection

Data were collected retrospectively from a combination of electronic patient records (EPRs) and paper clinical notes. The outcome and process measures extracted included the presence of a completed TEP, defined as documentation of a TEP at any point during admission; the time to TEP completion, measured in hours from hospital admission until the TEP form was signed; the presence of a documented DNACPR decision, recorded in the clinical notes; and the involvement of a senior clinician review, specifically whether a consultant geriatrician had signed or reviewed the TEP during admission. A data collection template was developed to ensure consistency across cycles, and all information was independently verified by two members of the QI team to minimize transcription errors.

Interventions

PDSA Cycle 1 (April 2025)

**Figure 1:**
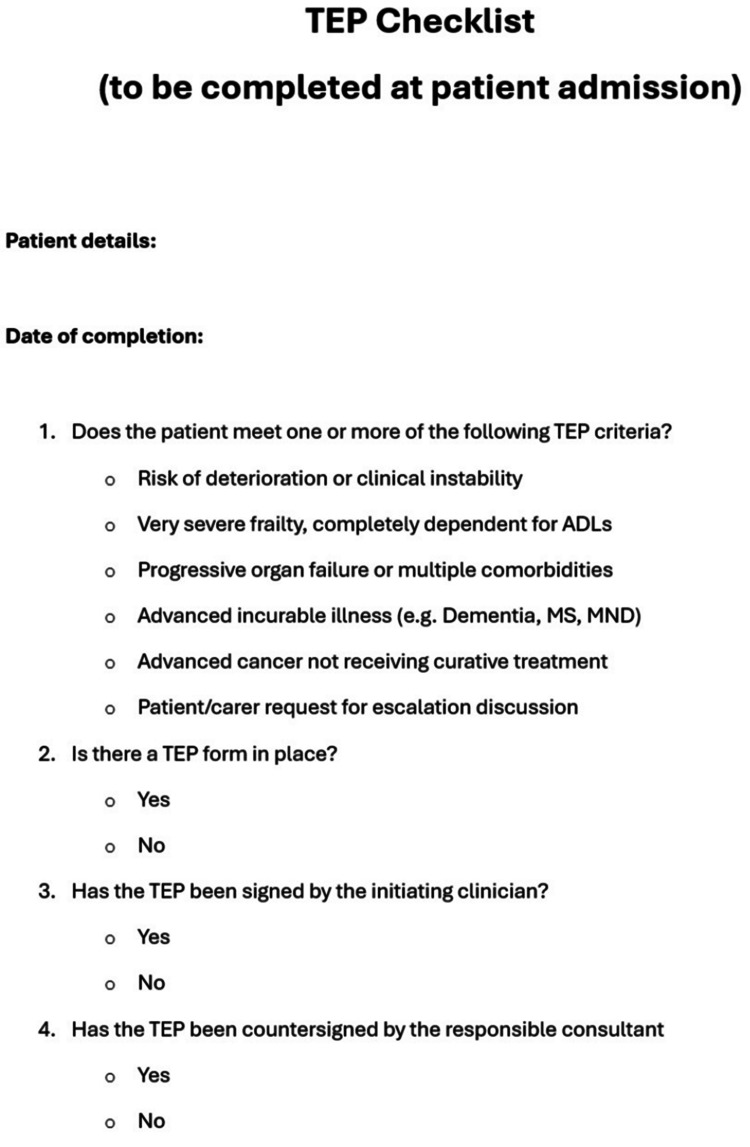
TEP checklist. TEP, treatment escalation plan; ADL, activities of daily living; MS, multiple sclerosis; MND, motor neuron disease

Data were collected on 75 consecutive eligible patients admitted to several geriatric medicine wards over one month. Following baseline analysis, a paper-based checklist was introduced as the first intervention (Figure 1). This checklist was inserted into patient admission notes and served as a prompt to ensure that a TEP was completed within 48 hours of admission, DNACPR decisions were documented, and senior clinician input was obtained.

PDSA Cycle 2 (June-August 2025)

This involved scaling the intervention hospital-wide across all geriatric wards. A structured multidisciplinary teaching session was delivered during departmental teaching and was attended by junior doctors, nursing staff, and allied health professionals. The session focused on the purpose and clinical application of TEPs, eligibility criteria and triggers for TEP initiation, principles of shared decision-making and patient-centered communication, relevant legal and ethical considerations in Scotland, and documentation standards including use of the standardized TEP form. Data were collected from 125 consecutive eligible patients admitted during these three months to evaluate the impact of the intervention.

Data analysis

All data were anonymized and entered into a secure spreadsheet using Microsoft Excel. Descriptive statistics were used to summarize patient demographics and process/outcome measures. Comparative analysis between PDSA cycles was performed using chi-squared tests for categorical data. A *P*-value <0.05 was considered statistically significant.

## Results

A total of 275 patients meeting TEP criteria were included across baseline and two PDSA cycles. The baseline cohort comprised 75 patients (70% female; mean age 80 ± 5 years), Cycle 1 included 75 patients (67% female; mean age 82 ± 7 years), and Cycle 2 comprised 125 patients (65% female; mean age 83 ± 8 years).

Following targeted interventions, TEP completion rates improved from 28% at baseline to 43% after Cycle 1, and further to 58% after Cycle 2. Median time to TEP completion decreased from 10.5 days (IQR 7-14) at baseline to 5 days (IQR 3-8) in Cycle 2. A summary of key outcomes is presented in Table 1.

**Table 1:** Summary of outcomes across baseline and Plan-Do-Study-Act (PDSA) cycles. TEP, treatment escalation plan; IQR, interquartile range; DNACPR, Do Not Attempt Cardiopulmonary Resuscitation

Outcome measure	Baseline (*n* = 75)	Cycle 1 (*n* = 75)	Cycle 2 (*n* = 125)
TEP completion, *n* (%)	21 (28%)	32 (43%)	72 (58%)
Median time to TEP (IQR)	10.5 days (7-14)	7 days (4-10)	5 days (3-8)
DNACPR documented, *n* (%)	41 (55%)	49 (65%)	91 (73%)
Consultant involvement, *n* (%)	9 (12%)	14 (19%)	34 (27%)

The proportion of patients with documented DNACPR decisions increased from 55% at baseline to 73% in Cycle 2, with a statistically significant association between DNACPR documentation and TEP completion (*P* = 0.01). Consultant involvement increased from 12% at baseline to 27% in Cycle 2 and was associated with shorter TEP completion times (median 5 vs. 7 days; *P* = 0.03).

Further analysis showed a statistically significantly higher TEP completion rate among patients aged ≥80 years compared to those aged 65-79 years (74% vs. 68%, *P* = 0.04), with no significant difference by gender (female 69% vs. male 67%, *P* = 0.68). A non-significant trend toward shorter hospital stays was observed in patients with timely TEP completion (<48 hours, *P* = 0.08).

## Discussion

Contemporary models of healthcare delivery emphasize patient-centeredness, recognizing individuals as active participants in their care. Within this framework, TEPs are essential tools that facilitate early, individualized discussions between patients and clinicians about appropriate levels of care in the event of deterioration. These conversations should address the risks, benefits, alternatives, and likely outcomes of interventions within the broader context of a patient’s health status, personal values, and treatment goals. Effective communication, tailored, honest, and compassionate is central to this process, enabling patients and, where appropriate, families or legal proxies to make informed decisions. Prior research underscores that early, transparent conversations reduce anxiety and improve satisfaction among patients and families [10]. Conversely, reactive decisions made during clinical crises are more likely to result in distress, decisional conflict, and misaligned care.

TEPs are particularly pertinent in geriatric medicine care, where frailty, multimorbidity, and cognitive impairment complicate prognostication and escalation decisions [11]. Our audit found higher TEP completion rates in patients aged ≥80 years, reflecting a growing awareness of the importance of anticipatory planning in this population. Older adults frequently experience episodic deteriorations superimposed on chronic illness, and in the absence of documented preferences, decisions are often made by on-call clinicians unfamiliar with the patient's history. TEP helps mitigate these risks by ensuring that care decisions remain consistent, proportionate, and in keeping with patient wishes, even out of hours.

Our study demonstrated an increase in TEP completion from 28% to 58%. This improvement is comparable to the findings of both Pattnaik et al. [3] and Chauhan et al. [12], who also observed a significant rise in TEP documentation following departmental awareness interventions. Both studies suggest that simple, structured interventions embedded within routine care pathways can effectively enhance timely decision-making documentation.

The concurrent rise in DNACPR documentation suggests that clearer escalation planning may reduce the likelihood of inappropriate or burdensome interventions. Without explicit guidance, clinicians may default to maximal interventions, even when they are unlikely to be beneficial. TEPs help define treatment ceilings, minimize moral distress among staff, and provide legal clarity by documenting decisions aligned with patient consent rather than ad hoc judgment [13]. They also support interprofessional collaboration by reducing ambiguity and conflict around escalation plans.

Beyond individual care, TEPs contribute to broader system efficiency. By supporting timely, well-informed decisions, they help reduce potentially avoidable ICU admissions and resource-intensive interventions for patients unlikely to benefit [14]. Although our audit found only a non-significant trend toward shorter hospital stays with early TEP completion, previous studies suggest that timely advance care planning may reduce hospital length of stay and readmission rates [15]. Further investigation with larger cohorts and longer follow-up is warranted to clarify the impact on clinical outcomes and resource utilization.

Despite measurable improvements following our interventions, TEP completion rates remained below ideal levels. Barriers such as time constraints, variability in clinician engagement, and documentation issues persist. Resistance may also arise from discomfort in discussing prognosis or uncertainty about patient preferences. Our multidisciplinary teaching intervention appeared effective in addressing knowledge gaps and promoting a culture supportive of shared decision-making. However, ongoing efforts such as embedding TEP prompts into electronic health records and continuous staff education are crucial to sustain progress.

Limitations of this quality improvement project include its single-center design and the relatively small sample size, which may affect generalizability. Retrospective data collection was dependent on the completeness of clinical documentation, potentially underestimating undocumented conversations. Additionally, our evaluation focused on process outcomes; we did not assess downstream effects such as patient or family satisfaction, quality of life, or treatment appropriateness. Future studies should incorporate qualitative feedback and explore longer-term clinical outcomes to provide a more comprehensive evaluation.

## Conclusions

This quality improvement project demonstrated that simple, targeted interventions such as admission checklists and multidisciplinary education can significantly enhance the uptake and timeliness of TEP in geriatric medicine settings. TEPs are vital tools for promoting patient autonomy, preventing non-beneficial interventions, and supporting consistent, informed decision-making during clinical deterioration. Sustaining these improvements will require system-level integration, ongoing staff engagement, and alignment with patient values. Future work should explore the impact of early TEP implementation on patient-centered outcomes and healthcare resource utilization.
